# Supplementary material for: Efficacy of DNA Vaccines in Protecting Rainbow Trout against VHS and IHN under Intensive Farming Conditions
Source: Vaccines (Basel). 2022 Dec 1;10(12):2062. doi: 10.3390/vaccines10122062 (PMC9780997; doi:10.3390/vaccines10122062)
Supplement: Supplementary file 1 [file vaccines-10-02062-s001.zip › Figure S1.pdf]

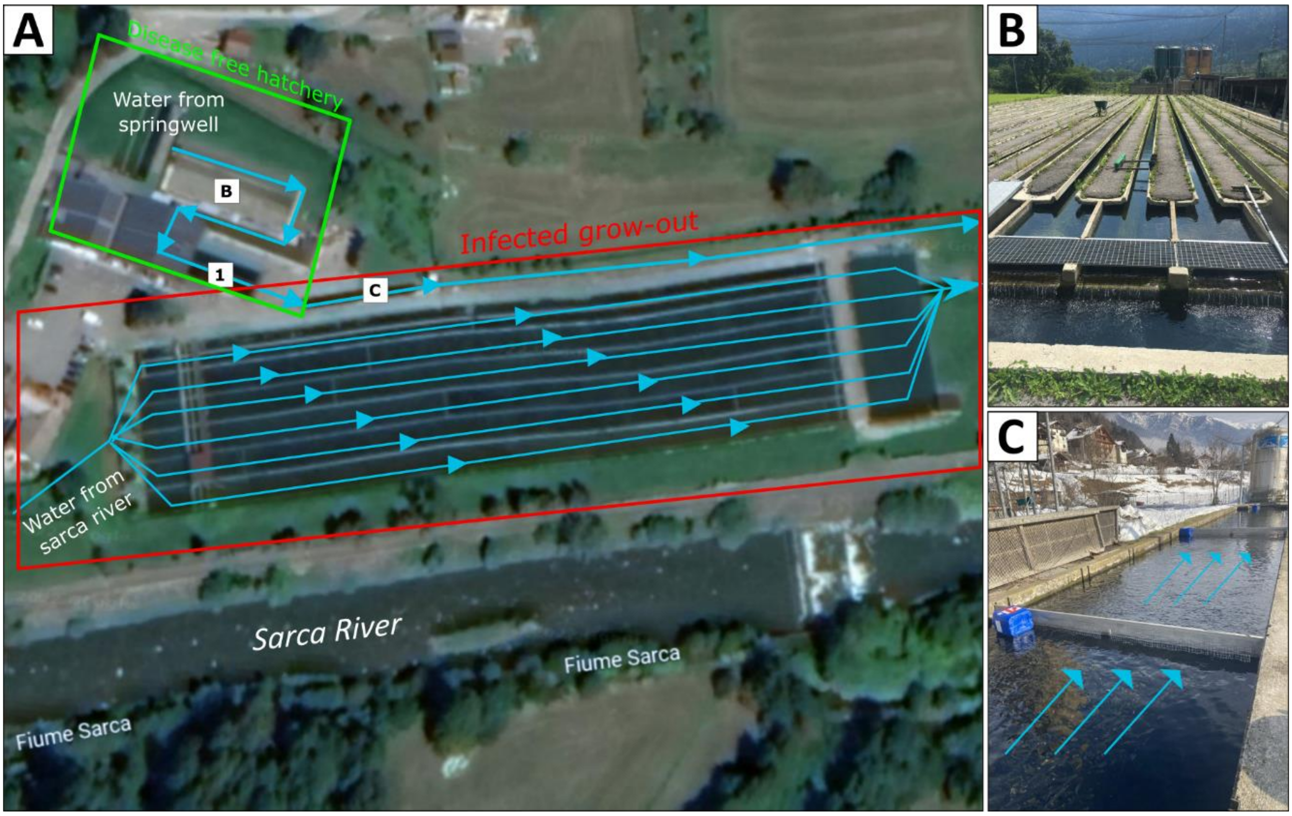

Figure S1: Field trial experimental setup. A) layout of the disease free hatchery (green romboid) and infected grow out (red romboid). Water paths are highlighted by the light blue lines. Position of the raceways housing fish destined to the slaughterhouse is reported (1). B) raceways housing experimental groups for 60 dpv in the disease free hatchery. C) Positioning of the experimental groups into the raceways. According to water flow, the mock vaccinated group was positioned first, followed by the group vaccinated against VHS. The VHS and IHN vaccinated group was positioned as last.
