# Supplementary material for: Efficacy of DNA Vaccines in Protecting Rainbow Trout against VHS and IHN under Intensive Farming Conditions
Source: Vaccines (Basel). 2022 Dec 1;10(12):2062. doi: 10.3390/vaccines10122062 (PMC9780997; doi:10.3390/vaccines10122062)
Supplement: Supplementary file 1 [file vaccines-10-02062-s001.zip › Figure S2.pdf]

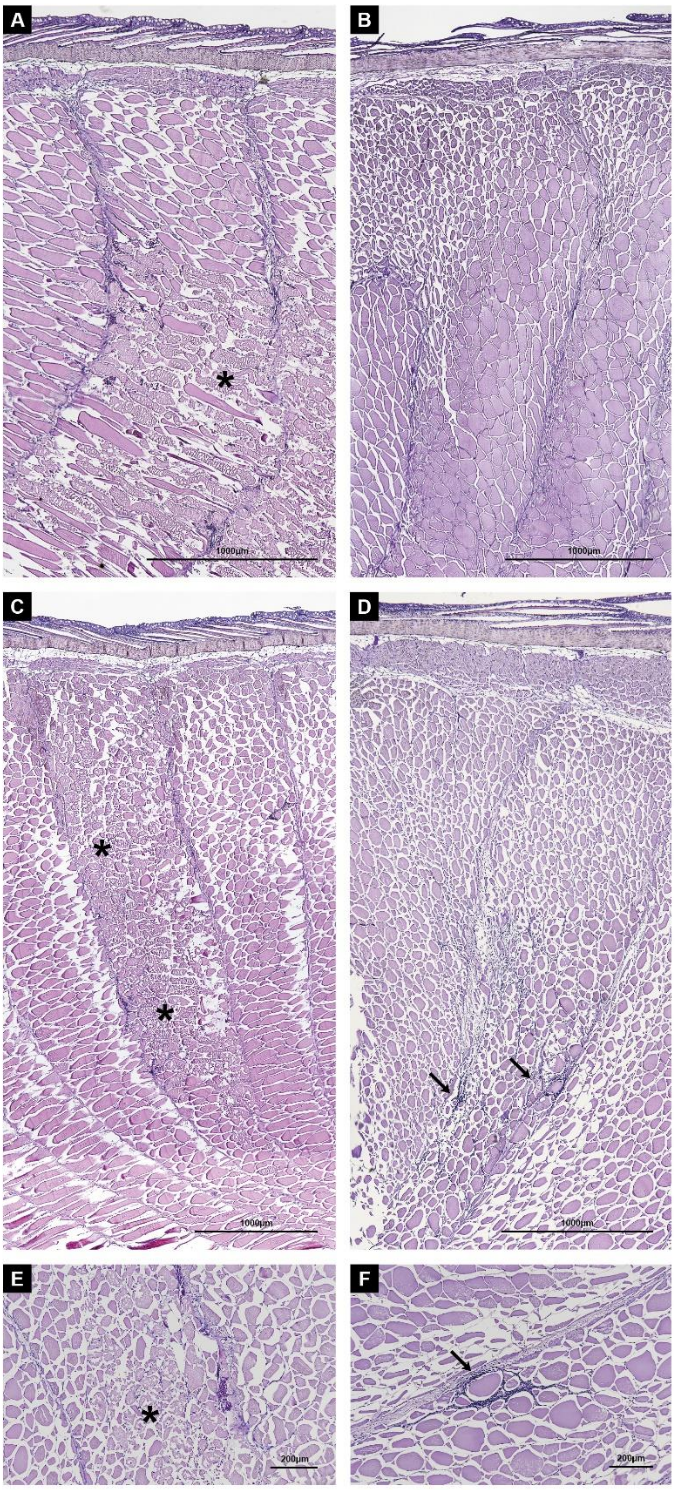

Figure S2: Histological analysis of muscle samples at different time points after vaccine administration and comparison with mock vaccinated controls, haematoxylin and eosin staining. A) epaxial muscle around the injection site of mock vaccinated specimen after 24 hpv; focal necrosis of muscle fibers is present (*), 4X B) epaxial muscle around the injection site of mock vaccinated specimen after 30 dpv; no fibrosis or inflammation is observable, 4X. C) epaxial muscle around the injection site of vaccinated specimen (1 µg/fish pVax-vhsG) after 24 hpv; focal necrosis of muscle fibers is present (*), 4X. D) epaxial muscle around the injection site of vaccinated specimen (1 µg/fish pVax-vhsG) after 30 dpv; inflammatory cells are present around muscle fibers (arrows), 4X. E) epaxial muscle around the injection site of vaccinated specimen (1 µg/fish pVax-vhsG) after 48 hpv; focal necrosis of muscle fibers is present (*) along with limited haemorrhages, 10X. F) epaxial muscle around the injection site of vaccinated specimen (1 µg/fish pVax-vhsG) after 30 dpv; inflammatory cells are present around muscle fibers (arrows), 10X.
