# Supplementary material for: Efficacy of DNA Vaccines in Protecting Rainbow Trout against VHS and IHN under Intensive Farming Conditions
Source: Vaccines (Basel). 2022 Dec 1;10(12):2062. doi: 10.3390/vaccines10122062 (PMC9780997; doi:10.3390/vaccines10122062)
Supplement: Supplementary file 1 [file vaccines-10-02062-s001.zip › Figure S3.pdf]

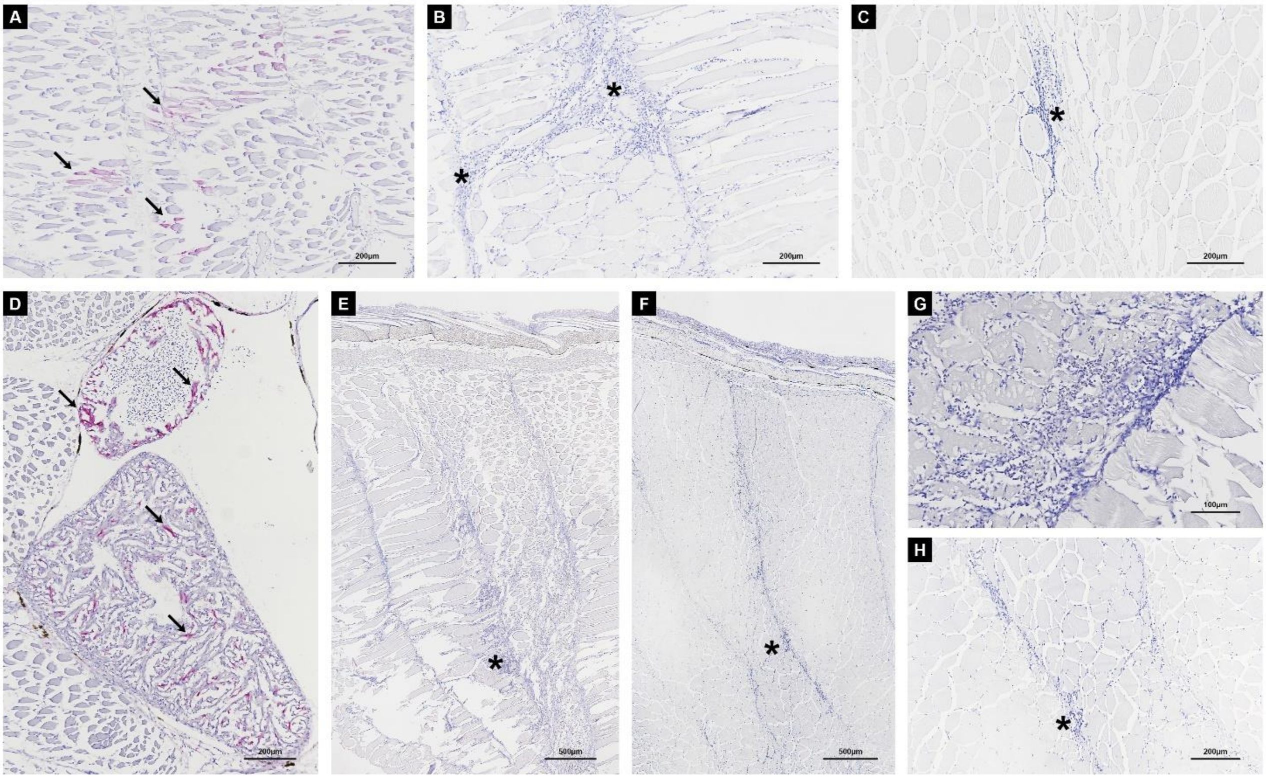

Figure S3: Histological analysis of muscle samples at different time points after VHS and IHN vaccine administration and comparison with infected VHS or IHN controls, IHC staining. A) epaxial muscle of moribund VHS infected specimens (challenged via immersion infection) showing IHC staining of affected fibers (arrows), 10X. B) epaxial muscle around the injection site of vaccinated specimen (2 μg/fish pVax-vhsG) for safety test after 15 dpv; inflammatory cells are present between muscle fibers but no IHC staining is observed, 10X. C) epaxial muscle around the injection site of vaccinated specimen (1 μg/fish pVax-vhsG) after 30 dpv; inflammatory cells are present between muscle fibers but no IHC staining is observed, 10X. D) heart of moribund IHN infected specimen (challenged via bath infection) showing IHC staining of affected cardiomyocytes (arrows), 10X. No IHC staining was observed in the skeletal musculature in this IHN affected specimen. E) epaxial muscle around the injection site of vaccinated specimen (2 μg/fish pVax-ihnG) for safety test after 15 dpv; inflammatory cells are present between muscle fibers but no IHC staining is observed, 4X. F) epaxial muscle around the injection site of vaccinated specimen (1 μg/fish pVax-ihnG) after 30 dpv; inflammatory cells are present between muscle fibers but no IHC staining is observed, 4X. G) detail of epaxial muscle of vaccinated specimen (2 μg/fish pVax-ihnG) for safety test after 15 dpv with abundant inflammatory cell presence, 20X. H) detail of epaxial muscle of vaccinated specimen (1 μg/fish pVax-ihnG) after 30 dpv with inflammatory cell presence, 10X.
