# Supplementary material for: Efficacy of DNA Vaccines in Protecting Rainbow Trout against VHS and IHN under Intensive Farming Conditions
Source: Vaccines (Basel). 2022 Dec 1;10(12):2062. doi: 10.3390/vaccines10122062 (PMC9780997; doi:10.3390/vaccines10122062)
Supplement: Supplementary file 1 [file vaccines-10-02062-s001.zip › Figure S4.pdf]

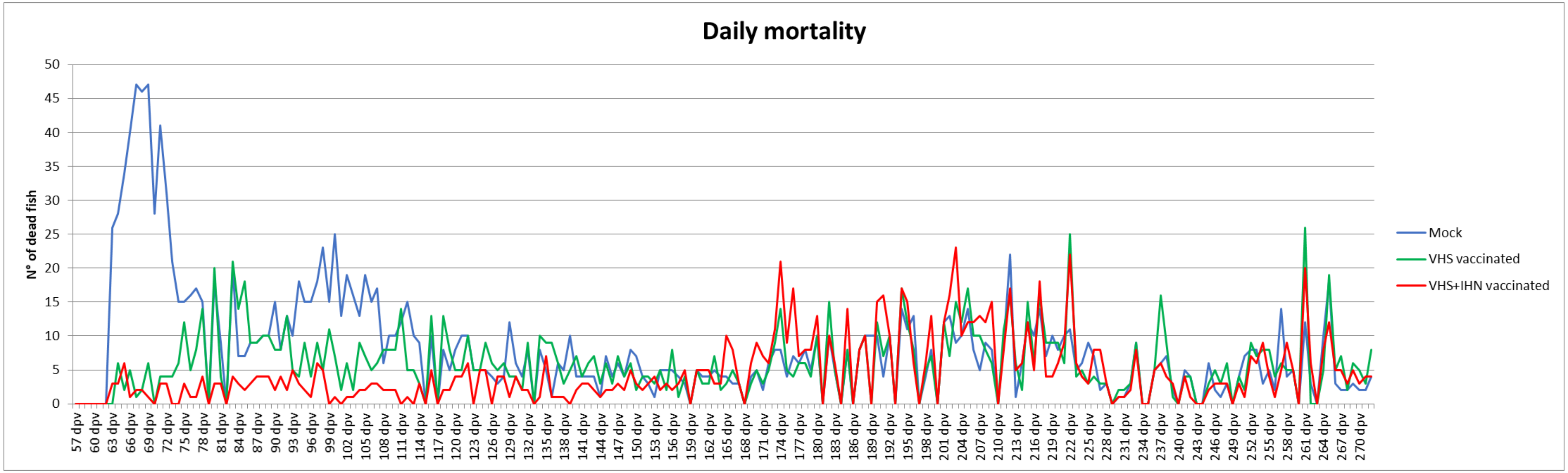

Figure S4: Daily mortality observed during the field trial. Mortality in the mock vaccinated group is represented by the blue line. Mortality in the VHS vaccinated group is represented by the red line. Mortality in the VHS+IHN vaccinated group is represented by the green line.
